# Sibutramine-induced mania as the first manifestation of bipolar disorder

**DOI:** 10.1186/1471-244X-12-43

**Published:** 2012-05-18

**Authors:** Napoleon Waszkiewicz, Beata Zalewska-Szajda, Sławomir Dariusz Szajda, Katarzyna Simonienko, Anna Zalewska, Agata Szulc, Jerzy Robert Ładny, Krzysztof Zwierz

**Affiliations:** 1 Department of Psychiatry, Medical University of Białystok, 16-070,, Choroszcz, Poland; 2 Department of Imaging Diagnostics, Medical University of Białystok, Children Hospital, Białystok, Poland; 3 Department of Emergency Medicine and Disasters, Medical University of Białystok, Białystok, Poland; 4 Department of Paedodontics, Medical University of Białystok, Białystok, Poland; 5 Medical College of the Universal Education Society, Łomża, Poland

**Keywords:** Obesity, Sibutramine, Mania, Bipolar disorder

## Abstract

**Background:**

Sibutramine, used in obesity treatment, has been associated with many neuropsychiatric side effects including hypomanic and manic episodes. Hypomanic/manic episodes related to sibutramine treatment were earlier reported in patients who had previous history of bipolar disorder, after sibutramine overdose, after over-the-counter product illegally containing very high dose of sibutramine, together with psychotic symptoms, in organic patient, or after interaction of sibutramine with other drugs.

**Case presentation:**

We report the first case of a patient with clear manic episode, after treatment with recommended dose of sibutramine, without previous history of mood disorders, organic changes or drug interactions, that was followed by episode of depression.

**Conclusion:**

Minimal recommended dose of sibutramine induced manic episode that was the first manifestation of bipolar disorder. The manic episode, associated with sibutramine treatment, was induced in a person without previous history of mood disorders. Potential risks associated with the treatment of obesity using sibutramine warn physicians to be alert not only to common and cardiovascular but also to psychiatric adverse effects. A careful assessment of patient’s mental state and detailed psychiatric family history should be done before sibutramine treatment. In patients with a family history for bipolar disorder the use of even minimal dose of sibutramine should be contraindicated.

## Background

Sibutramine, used in obesity treatment, is centrally acting serotonin-, norephiephrine-, and, to a lesser extent, dopamine- reuptake inhibitor [1]. It works by inducing satiety and thermogenesis. The anorectic effect of sibutramine and its metabolites is thought to be mediated via α_1-_ and β_1_-adrenergic as well as serotoninergic (5-HT_2B/2C_) receptors. The thermogenic effect of sibutramine is thought to be mediated by stimulating β_3_ adrenoreceptors in brown adipose tissue [2]. Sibutramine treatment has been associated with side effects: common (insomnia, nausea, dry mouth, and constipation), cardiovascular (increased risk of stroke and heart attack) and neuropsychiatric [2,3]. Case reports of neuropsychiatric disorders linked to the use of sibutramine include episodes of psychosis [4-6], affective psychosis [7-9], panic attacks [10], depression with suicidal tendencies [1], delirious state [11], amnesia [12], and hypomanic or manic episodes [13-15]. While on sibutramine medication, psychiatric episodes needed 3 to12 weeks to develop for psychosis, more than 10 days for panic attacks, more than 2 weeks for delirious and hypomania/mania states, and from 1 day to 1 month for amnesia episodes. During sibutramine treatment manic and hypomanic episodes were earlier reported in patients who had: recommended-daily-dose of sibutramine (up to 15 mg/day) and previous history of bipolar disorder [13,14], an organic hypomanic episode secondary to sibutramine-citalopram interaction (10 mg/day of sibutramine) [15], mixed episode after sibutramine overdose (30 mg/day) [16], recurrent affective psychosis after the therapeutic dose of sibutramine [8] or de novo affective psychosis in relation to sibutramine-sertraline interaction [7] and after over-the-counter product illegally containing therapeutic or above the normal range doses of sibutramine (2-3 fold higher than recommended dose) [9,17]. A Medline search failed to find any report of clear manic episode triggered by recommended dose of sibutramine medication, in person without previous history of mood disorders, organic brain changes or drug interactions.

## Case report

A 23-year-old woman was brought to the psychiatric department by her mother because of a change in behavior that appeared four weeks before the submission. At submission day (sixth week of sibutramine treatment at the recommended dosage of 10 mg/day), she presented elevated and expansive mood, increased energy and explosive reactions, was talkative and reported racing thoughts. Her mother stated that daughter had not slept for the past 4 weeks but felt rested, and stayed up all night cleaning the house. Daughter speech was rapid and loud, and it was hard to interrupt her. Mother said that daughter was out of home overnight on two occasions in the past month, being picked up by police for public alcohol drinking. It was behavior very unlikely her usual self. The patient stated that she just wanted to get out of the house to visit her friends and meet new interesting people. Following sibutramine withdrawal and introduction of valproic acid (2000 mg/day), the patient experienced a remission of manic episode in approximately 2 weeks.

Six weeks later, she was admitted to the psychiatric department again with complaints of a depressed mood, loss of interest and pleasure, and marked psychomotor retardation. She said that for more than the past 2 weeks she often stayed in bed all day because of fatigue and lack of motivation. She felt guilty about the irresponsibility and excesses of the previous manic episode. She could not sleep and woke up early morning, stopped eating and bathing, her ability to think and concentrate diminished markedly. Before the manic episode, there was no significant past medical, psychiatric or substance abuse history. The results of the patient’s physical and neurological examination, laboratory tests (including function of thyroid, liver and kidney), magnetic resonance imaging (MRI) of her brain, and ECG did not reveal any significant abnormalities. She had a positive family history for depression in her grandmother and bipolar disorder in her aunt. She was diagnosed as having a bipolar disorder, most recent episode depressed. In the psychiatric department, valproic acid treatment was maintained at the dose of 1500 mg/day, and sertraline 100 mg/day was started, with good results. In view of significant improvement, the patient was discharged after one month of hospitalization. There were no symptoms of mood disorder during 1 year of follow-up.

## Conclusion

The temporal relationship between sibutramine intake and onset of behavioral changes as well as the decrease in manic symptoms with cessation of sibutramine and initiation of anti-manic pharmacotherapy led us to suspect the role of sibutramine in the pathogenesis of manic episode. It could be hypothesized that sibutramine may induce mania in predisposed individuals by acting in a similar way with other selective serotonine reuptake inhibitors (SSRIs) [14]. In the 1980s sibutramine was initially intended as an antidepressant drug [3]. In the 2000s antidepressant-associated mania has been linked to all major antidepressant classes in a subgroup of 20-40 % of bipolar patients [18,19]. Bipolar spectrum incorporates classic bipolar disorder (manic + depressive episode), bipolar II disorder (hypomanic + depressive episode), and bipolar III disorder that is not an official diagnosis recognized by psychiatric associations. Although we will not find bipolar III disorder mentioned in the International Classification of Diseases (ICD-10) or Diagnostic and Statistical Manual of the American Psychiatric Association (DSM-IV), psychiatric professionals use the unofficial diagnosis of bipolar III disorder to describe patients who have experienced manic or hypomanic episode due to antidepressant treatment [20]. The serotonin transporter gene is a candidate to be associated with antidepressant-associated mania in some patients [21]. The serotonin transporter gene demonstrates a polymorphism within the promoter region (5-HTTLPR) with two allelic forms -the long and the short variants. Since 5-HTTLPR polymorphism is considered as a predictor of abnormal response to antidepressants in vulnerable to bipolar disorder patients, a correct diagnosis of bipolarity and detailed family history for affective disorders should be done before the beginning of sibutramine treatment. It is essential mainly for short variant of 5-HTTLPR carriers [19]. As chronic sleep deprivation can precipitate the manic episode, insomnia (a very common side-effect of sibutramine) might exacerbate manic symptoms in our vulnerable patient. It was also hypothesized that individuals can engage in overeating for the purpose of regulating their mood [22]. Therefore, bipolar patients may overeat to “self medicate” their affective symptoms, and suppression of overeating and/or weight loss might therefore trigger the onset or exacerbation of the mood symptoms. It is also possible that the weight loss might also be associated with release of toxins, including drugs that might have mood destabilizing effects [22].

Although the neuropsychiatric side effects of sibutramine have not yet been completely elucidated, there is accumulating literature on a variety of psychiatric disturbances caused by sibutramine. Further studies are required to clarify the relationship between sibutramine and episodes of psychiatric disorders including mania. Sibutramine, as an antiobesity drug was approved in the USA in 1997 and in the European Union in 1999, but recently followed the example of another antiobesity drug -rimonabant (an inverse agonist for the cannabinoid receptor CB1) that was removed from all markets in the European Union [23]. There was concluded that the risks of cardiovascular complications of sibutramine were greater than its benefits. Potential risks associated with the sibutramine treatment of obesity warn physicians to be alert not only to cardiovascular events but also to psychiatric adverse effects. Therefore a careful assessment of patient’s mental state together with detailed family psychiatric history should be done before beginning sibutramine treatment. If patients present a first episode of psychosis, mania, or other psychiatric disorder, they should be investigated thoroughly while sibutramine is being withheld [3]. It is important to obtain information about vulnerability of the patient to psychiatric disorders and whether eventual symptoms were due to a primary psychiatric disorder or to a drug-induced episode. In patients with weight gain while on antipsychotic medications (e.g. olanzapine treatment), sibutramine, if not managed appropriately, could conceivably exacerbate psychotic episode symptoms or trigger its relapse. Physicians should also remember that use of sibutramine is contraindicated not only in patients with a history of coronary artery disease, heart failure, arrhythmias, cerebrovascular disease, inadequately controlled hypertension, bulimia/anorexia nervosa, pregnancy, lactation, severe renal or liver dysfunction, narrow angle glaucoma, but also in patients treated with monoamineoxidase inhibitors (MAOIs), selective serotonine reuptake inhibitors (SSRIs) or certain migraine drugs as triptans, because of potential risk of serotonin syndrome. Thus, a 2-week interval is required after stopping MAIOs -before treatment with sibutramine, and after stopping sibutramine -before treatment with MAOIs [3].

All of the abovementioned aspects of sibutramine treatment require careful weighting, and benefits of obesity pharmacotherapy must outweigh the risks and costs. Albert Einstein said: “The devil has put a penalty on all things we enjoy in life. Either we suffer in health or we suffer in soul or we get fat”, but patients do not have to suffer in health or soul when they try to lose fat. When the patient has a positive family history for bipolar disorder, a change to the other antiobesity drug than sibutramine may be required (e.g. to orlistat). The neurobiology of obesity is extremely complex and many novel potential antiobesity drugs and targets are identified including those acting on the central pathways as ciliary neurotrophic factor, melanocortin-4 receptor agonists, ghrelin, neuropeptide Y antagonists, melanin-concentrating hormone antagonists, or peptide Y [2,3]. However, the most effective long-term weight loss still depends on motivation for permanent decrease in energy intake, improving dietary quality, and increase in physical activity. Further research should take into consideration the use of a mood stabilizers in prevention of mood changes in predisposed individuals, before sibutramine treatment. The main conclusion of our case is that the use of sibutramine (even in minimal recommended dose) should be contraindicated in patients with a family history for bipolar disorder.

## Competing interests

The author(s) declare that they have no competing interests.

## Authors' contribution

NW conceptualized, treated and followed up the patient and wrote the case report. BZS did neuroimaging. SDS conceptualized, did laboratory measurements. KS conceptualized, did literature survey. AZ conceptualized, did literature survey. AS treated the patient and helped in report writing. JRŁ did literature survey, conceptualized. KZ conceptualized helped in report writing. All authors read and approved the final document.

## Pre-publication history

The pre-publication history for this paper can be accessed here:

http://www.biomedcentral.com/1471-244X/12/43/prepub
